# Variation of Human Salivary O-Glycome

**DOI:** 10.1371/journal.pone.0162824

**Published:** 2016-09-09

**Authors:** Radoslaw P. Kozak, Paulina A. Urbanowicz, Chamindie Punyadeera, Karli R. Reiding, Bas C. Jansen, Louise Royle, Daniel I. Spencer, Daryl L. Fernandes, Manfred Wuhrer

**Affiliations:** 1 Ludger Ltd., Culham Science Centre, Oxfordshire, United Kingdom; 2 School of Biomedical Sciences, Institute of Health and Biomedical Innovations, Queensland University of Technology, 60 Musk Avenue, Kelvin Grove, Australia; 3 Centre for Proteomics and Metabolomics Leiden University Medical Centre, Leiden, The Netherlands; University of Maryland, College Park, UNITED STATES

## Abstract

The study of saliva O-glycosylation is receiving increasing attention due to the potential of glycans for disease biomarkers, but also due to easy access and non-invasive collection of saliva as biological fluid. Saliva is rich in glycoproteins which are secreted from the bloodstream or produced by salivary glands. Mucins, which are highly O-glycosylated proteins, are particularly abundant in human saliva. Their glycosylation is associated with blood group and secretor status, and represents a reservoir of potential disease biomarkers. This study aims to analyse and compare O-glycans released from whole human mouth saliva collected 3 times a day from a healthy individual over a 5 days period. O-linked glycans were released by hydrazinolysis, labelled with procainamide and analysed by ultra-high performance liquid chromatography with fluorescence detection (UHPLC-FLR) coupled to electrospray ionization mass spectrometry (ESI-MS/MS). The sample preparation method showed excellent reproducibility and can therefore be used for biomarker discovery. Our data demonstrates that the O-glycosylation in human saliva changes significantly during the day. These changes may be related to changes in the salivary concentrations of specific proteins.

## Introduction

Saliva is a unique fluid that lubricates and buffers the oral cavity [1–2]. It protects the oral cavity from microbial attack involving both the innate and acquired immune surveillances. Saliva is a structurally very complex secretion containing components from a variety of glands such as the parotid, submaxillary, sublingual and submandibular glands and some minor glands in the lip, cheek, tongue and palate [3]. Saliva contains a wide spectrum of microbes, epithelial cells, proteins/peptides, electrolytes, hormones, nasal and bronchial secretions and serum product [4–7].

Saliva contains many glycoproteins, some of which are highly glycosylated (such as MUC5B and MUC7) as well as salivary agglutinin, secretory immunoglobulin A (sIgA), lactoferrin, amylase and proline-rich glycoproteins [8]. Mucins are dominant compounds in human saliva and play an important role in the maintenance of oral health and protection of teeth as part of the first line oral defence [9–10]. Many glycoproteins bind to a variety of bacteria and either aid or prevent adherence of bacteria to mucosal and tooth surfaces or lubricate tooth surfaces and possibly protect proteins from proteolytic attack [11].

Bacteria usually have a net negative surface charge and therefore tend not to bind directly to negatively charged mucins. Instead, they may interact through bridging reagents involving divalent cations such as Ca^2+^ ions. However, many bacteria express cell surface proteins (lectins) that specifically recognize oligosaccharides present on the salivary mucins. In this manner salivary mucins are bound by multiple bacteria resulting in agglutination. Removal of the terminal sialic acids from the oligosaccharide side chains of salivary mucins was found to abolish agglutination of oral streptococci, indicating that the bacterial lectin has specificity for sialic acids [12]. Salivary agglutinin, a highly glycosylated protein frequently associated with other salivary proteins and with secretory immunoglobulin A (IgA), is one of the main salivary components responsible for bacteria agglutination [13].

Secretory IgA is the most abundant humoral immunologic component of saliva. It can neutralize viruses, bacteria, and enzyme toxins. It binds bacterial antigens and is able to aggregate bacteria and inhibit their adherence to oral tissues [14–15]. Other immunologic components, such as IgG and IgM, occur in lower quantity and probably originate from gingival fluid [16].

Salivary composition shows associations with various oral and systemic diseases [17–22], and significant age- or sex-related differences have been reported with respect to genomic [23, 24]and proteomic expression levels [25–27].

Recently, interest in glycomics (the study of the spectrum of glycans expressed in a biological system) has increased due to the potential clinical utility of glycans as disease biomarkers [28]. Glycosylation of proteins is a complex and common post-translational modification influencing many biological functions. There are two main types of glycosylation found in eukaryotes: N-glycosylation and O-glycosylation. On salivary glycoproteins, N-glycans are structurally diverse encompassing the high mannose, hybrid and complex N-glycan subclasses. The O-glycans comprise various O-glycan core types, with optionally fucoses in different linkages as well as sialylation and sulphation [29, 30]. It has been reported that salivary MUC5B contains O-glycans which vary between individuals and that some of this variation is related to blood group and secretor status [31]. Also O-glycans attached to salivary MUC7 showed changes in the patients with recurrent aphthous stomatitis (RAS). Patients with RAS have less complex O-glycans, suggesting that there are bacterial oligosaccharide-degrading enzymes that reduce glycan complexity [32].

Human saliva proteomics has proven to be a novel approach in the search for protein biomarkers for detection of diseases [31], including cancer, autoimmune diseases, viral diseases, bacterial diseases, cardiovascular diseases, and human immunodeficiency virus (HIV) [2, 33]. It has become clear that proteomic composition of saliva can reflect the pathological as well as physiological state [34, 35]. Recently, a growing number of proof-of-principle assays have been established using saliva to monitor diseases or bodily conditions such as infection, immune responses to viral infections, systemic levels of drugs, and the detection of illicit drug use [36]. Saliva profiling over the course of disease progression could reveal potential biomarkers indicative of different stages of diseases, which may be useful in medical diagnostics. This follows from several studies showing that glycosylation patterns in other body fluids such as plasma correlate with progression of certain diseases, including inflammatory conditions and subclasses of diabetes [37–39].

Here, we describe the evaluation of longitudinal changes in salivary O-glycosylation profiles using samples collected 3 times a day over a 5 days period. The O-glycans were released by hydrazinolysis using a recently developed improved protocol [40] and fluorescently labelled with procainamide, followed by HILIC-UHPLC-FLR-ESI-MS analysis. The chosen techniques proved to be suitable to demonstrate major changes in the salivary O-glycome during the day.

## Materials and Methods

### Materials

All reagents and kits for O-glycan release, labelling and clean-up were from Ludger Ltd. (Oxford, UK). Centrifugal filter devices with a molecular weight cut-off (MWCO) membrane of 10 kDa were obtained from Fisher Scientific UK (Leicestershire, UK). Acetonitrile (Romil; 190 SpS for UV/ gradient quality) was obtained from Charlton Scientific (Charlton, Oxon, UK). Fetuin glycoprotein was from Ludger Ltd and all other reagents were obtained from Sigma-Aldrich (Dorset, UK). Procainamide labelling was performed on a Hamilton Microlab STARLet liquid-handling robotic platform.

### Collection of saliva

Whole unstimulated saliva (5 mL) was collected 3 times a day over a 5 days period from two human donors. Volunteers were asked to rinse their mouths with water for 30 seconds to remove any food particles. Donors were also asked to retain from eating 2 h prior to collection [3]. PBS supplemented with protease inhibitor cocktail (2 mL, AEBSF at 2 mM, Aprotinin at 0.3 μM, Bestatin at 116 μM, E-64 at 14 μM, Leupeptin at 1 μM and EDTA at 1 mM) and antibiotic-antimycotic mixture containing 10,000 units penicillin, 10 mg streptomycin and 25 μg amphotericin B per mL were added to each saliva sample. The saliva samples were clarified by centrifugation at 3000 x g at 4°C for 20 min to remove cellular debris. The supernatant was concentrated [29] using centrifugal filter device (10 kDa MWCO membrane). Samples were then stored at -20°C until required for further experimentation.

### Release of O-glycans by hydrazinolysis

The saliva samples and fetuin glycoprotein (used as a positive control) were buffer exchanged into 0.1% TFA prior to hydrazinolysis as described previously [40]. Samples were transferred to glass vials and dried down for 16 h by vacuum centrifugation prior to the addition of hydrazine.

Fetuin and saliva samples were incubated with 450 μL anhydrous hydrazine (Ludger) at 60°C for 6 h [41, 42]. Excess hydrazine was removed by centrifugal evaporation. The samples were placed on ice for 20 min (0°C) and were re-*N*-acetylated by addition of 0.1 M sodium bicarbonate solution (200 μL) and acetic anhydride (21 μL). Samples were cleaned up by passing them through the Ludger Clean CEX cartridges (Ludger). The O-glycans were eluted off the cartridges using water (3 x 0.5 mL). Eluates were dried by vacuum centrifugation prior to fluorescent labelling.

### Procainamide labelling

Of the released O-glycans, 40% was fluorescently labelled with procainamide as described previously [43] using Ludger procainamide glycan labelling kit containing 2-picoline borane. Briefly, samples in 10 μL of water were mixed with 10 μL of procainamide labelling solution and incubated at 65°C for 1 h. The samples were evaporated to dryness by vacuum centrifugation and re-suspended in water (100 μL) for further analysis.

### LC-ESI-MS and MS/MS analysis

Procainamide labelled samples were analysed by HILIC-UHPLC-ESI-MS with fluorescence detection. Samples were injected in 10% water/90% acetonitrile (injection volume 25 μL) onto an ACQUITY UPLC BEH-Glycan 1.7 μm, 2.1 x 150 mm column at 60°C on a Ultimate 3000 UHPLC instrument with a fluorescence detector (λex = 310nm, λem = 370nm; Thermo Scientific). The running conditions used were: Solvent A was 50 mM ammonium formate pH 4.4 made from Ludger Stock Buffer (Ludger), and solvent B was acetonitrile. Gradient conditions were: 0 to 10 min, 90% B; 10 to 95 min, 90 to 57% B at flow rate 0.4 mL/min; 95 to 98 min, 57 to 0% B at a flow rate of 0.4 mL/min; 98 to 99 min, 0% B at a flow rate of 0.25 mL/min; 99 to 100 min, 0% B at a flow rate of 0.25 mL/min. 100 to 103 min, 90% B at a flow rate 0.25 mL/min; 103 to 115 min, 90% B at a flow rate 0.4 mL/min. The UHPLC system was coupled on-line to an Amazon Speed ETD (Bruker Daltonics, Bremen, Germany) with the following settings: source temperature 250°C, gas flow 10 L/min; Capillary voltage 4500 V; ICC target 200,000; maximum accumulation time 50 ms; rolling average 2; number of precursors ions selected 3, release after 0.2 min; Positive ion mode; Scan mode: enhanced resolution; mass range scanned, 200–1600; Target mass, 900. A glucose homopolymer ladder (Ludger), labelled with procainamide was used as a system suitability standard as well as an external calibration standard for GU allocation [44].

### Sample variation

Using in-house developed scripts, raw HILIC chromatograms for 2 individuals were exported from Chromeleon 7.1 (Dionex, USA) and aligned by applying the least variance linear fit through the highest data points observed within ± 0.25 min window around retention times 22.33, 27.13, 28.70, 29.28, 32.43 and 33.64 min. Then, for each chromatogram independently, the areas of the 7 most abundant peaks were integrated centering at 20.55 min (within a ± 0.5 min window), 22.26 (± 0.35), 23.99 (± 0.35), 24.74 (± 0.5), 27.17 (± 0.35), 32.39 (± 0.25) and 33.57 (± 0.25). The obtained peak areas were normalized to the total area sum within each chromatogram, and expressed in stripplot format using R version 3.1.2 (R Foundation for statistical computing, Vienna Austria).

### Ethics statement

The Research Ethic Committee (NHS Health Research Authority, Surrey Borders REC London, UK) granted approval for this study with all participants giving written, informed consent (REC 16/LO/0883; IRAS project ID: 203515).

## Results and Discussion

### Evaluation of method repeatability

The release and recovery of O-glycans still remains very challenging because there is no universal enzyme available for O-glycan release from glycoproteins. Therefore, the current best methods for universal O-glycan release rely on rather harsh chemistry. Over the last two decades a number of chemical methods for O-glycan removal have been developed, including reductive and non-reductive β-elimination and hydrazinolysis [45]. It has been reported that the most suitable method for universal O-glycan release in their non-reduced form in high yields is hydrazinolysis [41]. We decided to use this technique to release O-glycans from human saliva because the released O-glycans contain free reducing termini allowing further derivatization with fluorescent tags and analysis by HPLC-based methods [40, 46].

First, we investigated whether the sample preparation and glycan release method is suitable for saliva samples. Using fetuin glycoprotein and a saliva reference sample collected from a single individual as positive controls, we evaluated the O-glycan preparation and O-mode hydrazinolysis. Triplicates of bovine fetuin and saliva samples were buffer exchanged into 0.1% TFA prior to release [40]. The O-glycans were released using anhydrous hydrazine, labelled with procainamide and analysed by HILIC-UHPLC with fluorescence detection providing good glycan separation, coupled to ESI-MS and MS/MS providing glycan composition.

**Fig 1:**
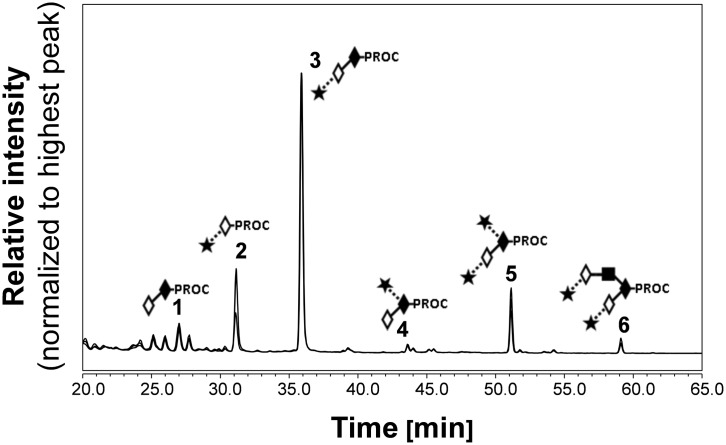
HILIC-UHPLC profiles for procainamide labelled O-glycans released in triplicate from fetuin. The following symbols are used to depict glycan structures [57]: open diamond = galactose; closed diamond = N-acetylgalactosamine; closed square = N-acetylglucosamine; closed star = N-acetyleneuraminic acid; dashed line, α-linkage; solid line, β-linkage.

**Fig 2:**
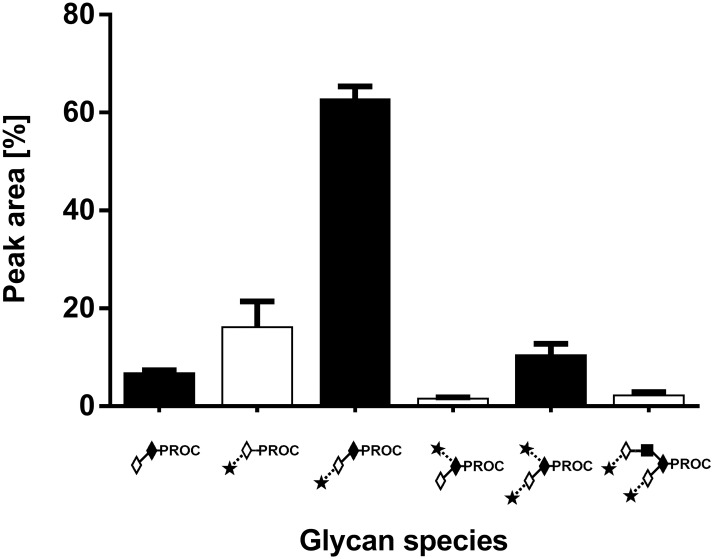
O-glycans patterns obtained from bovine fetuin. Samples were washed with 0.1% TFA followed by hydrazinolysis, procainamide labelling and HILIC-UHPLC analysis with fluorescence detection. The average abundance of the O-glycans was determined on multiple days. Symbols are as in Fig 1.

The HILIC-UHPLC profiles for triplicate releases of O-glycans from fetuin sample (Fig 1) were consistent with previously published data [46, 47]. The fetuin profiles contain 6 main peaks corresponding to: core 1, Galβ1–3GalNAc (peak 1); monosialylated core 1 O-glycans, Neu5Acα2–3Galβ1–3GalNAc (peak 3); and Neu5Acα2–6(Galβ1–3)GalNAc (peak 4); disialylated core 1 O-glycan, Neu5Acα2–3Galβ1–3(Neu5Acα2–6)GalNAc (peak 5); and disialylated core 2 O-glycan, Neu5Acα2–3Galβ1–3(Neu5Acα2–3Galβ1–4GlcNAcβ1–6)GalNAc (peak 6). The peeled product Neu5Acα2–3Gal (peak 2, Fig 1) was also detected. To confirm the identity of observed peaks (peaks 1–6, Fig 1) the procainamide labelled O-glycans were characterized by ESI-MS/MS. The results from HILIC-UHPLC coupled to ESI-MS/MS are summarised in S1 Table and an example of fragmentation analysis of procainamide labelled O-glycans is shown in S1 Fig. The glycan peak areas for each release were very similar with the standard deviation between the samples below 5.5% for all peaks (Fig 2).

**Fig 3:**
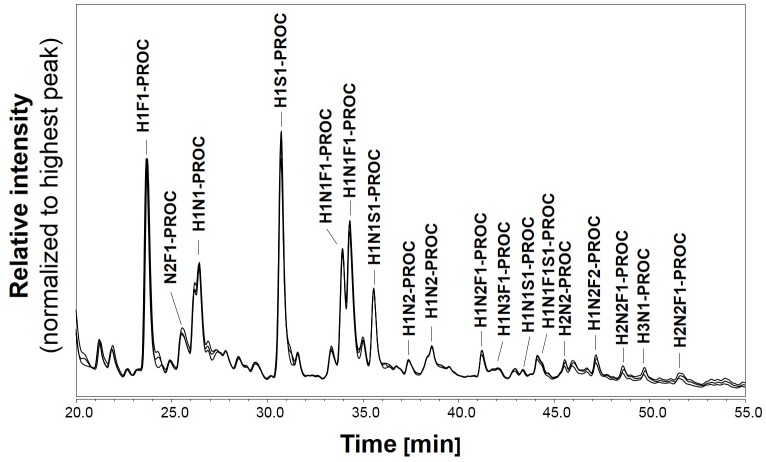
HILIC-UHPLC O-glycan profiles of saliva reference sample collected from a single individual in the morning. Sample was buffer exchange into 0.1% TFA, and the O-glycans were released in triplicate by hydrazinolysis followed by procainamide labelling and HILIC-UHPLC analysis with fluorescence detection.

**Fig 4:**
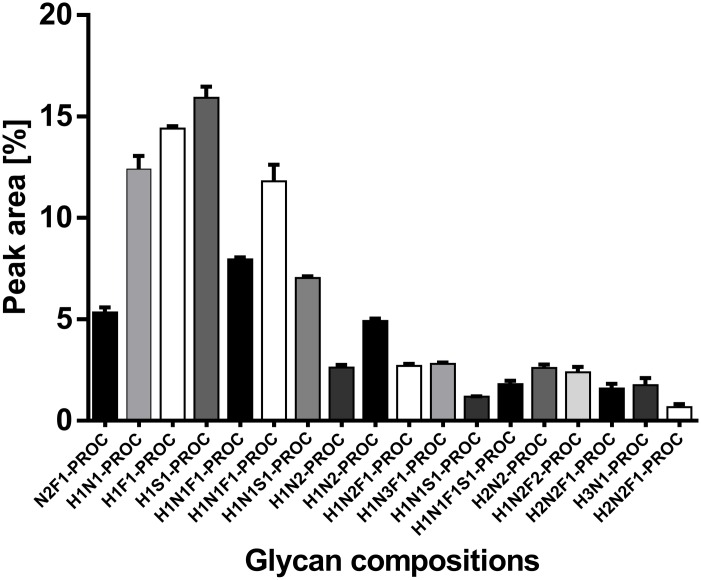
O-glycan patterns obtained from triplicate releases from saliva sample collected in the morning. Samples were washed with 0.1% TFA followed by hydrazinolysis, procainamide labelling and HILIC-UHPLC analysis with fluorescence detection. 18 O-glycan structures were selected for comparison.

The robustness of the O-glycosylation analysis method was also tested on the saliva standard. The O-glycans were released by anhydrous hydrazine, labelled with procainamide and analysed by HILIC-UHPLC-ESI-MS with fluorescence detection. The HILIC-UHPLC profiles for triplicate releases of O-glycans from whole saliva sample are shown in Fig 3. As the profiles were very complex we selected a subset of 18 peaks for comparison. These peaks were analysed by ESI-MS/MS to determine O-glycan composition. The glycan peak areas for selected peaks were very similar with the standard deviation between the samples below 1% (Fig 4). Thus, O-mode hydrazinolysis, procainamide labelling systems and UHPLC-ESI-MS/MS analysis are highly reproducible and suitable for the analysis of human saliva samples.

### Longitudinal analysis of O-glycans from human saliva

**Fig 5:**
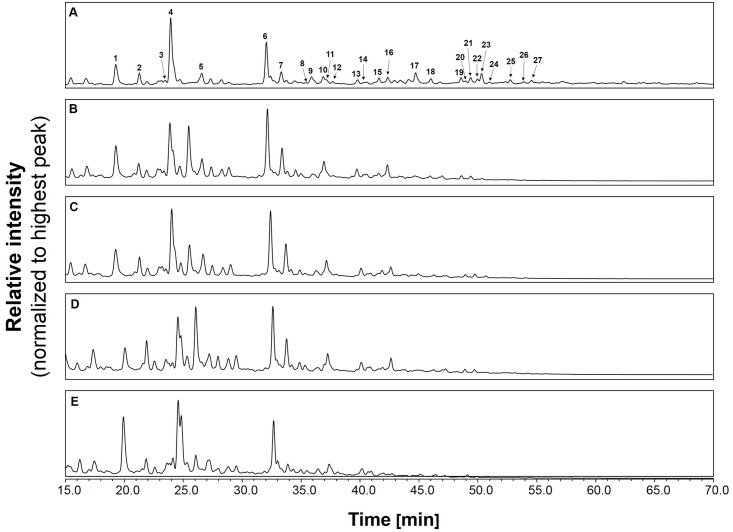
HILIC-UHPLC profile of saliva O-glycan patterns. Samples were collected in days 1–5 in the morning. Samples were buffer exchanged into 0.1% TFA, and the O-glycans were released by hydrazinolysis followed by procainamide labelling and HILIC-UHPLC analysis with fluorescence detection.

O-glycans were released from saliva (collected 3 times a day over a 5 days period from 2 individuals) by hydrazinolysis after acidic wash, labelled with procainamide and analysed by UHPLC-ESI-MS/MS. Only one sample was taken through ESI-MS/MS analysis. As the obtained HILIC profiles were very complex we selected a subset of 27 peaks for comparison and detailed analysis by ESI-MS/MS (S2 Fig). A broad range of O-glycan compositions were detected in the samples and although there was some overlap in LC traces, the sample profiles showed prominent differences. The observed molecular masses and compositions of 27 selected structures (Fig 5) are summarised in Table 1.

**Table 1:** Mass spectrometric data for selected 27 O-glycans obtained from human saliva.

UHPLC Peak Id	Average GU (PROC)	HILIC-UHPLC-ESI-MS						
		Composition				Calculated [m/z]^+^	Registered [m/z]^+^	Probable structures
		Hex (H)	HexNAc (N)	Fucose (F)	Neu5Ac (S)			
1	1.40	0	1	1	0	587.38	587.32	-
2	1.51	1	0	1	0	546.38	546.30	F-H-PROC
3	1.63	0	2	1	0	791.45	790.41	F-N-N-PROC
4	1.66	1	1	0	0	603.39	603.32	H-N-PROC
5	1.81	1	1	1	0	749.45	749.38	F-H-N-PROC
6	2.21	1	1	1	0	749.45	749.38	F-H-N-PROC
7	2.30	1	1	0	1	894.48	894.42	S-H-N-PROC
8	2.45	1	2	0	0	806.46	806.40	N-(H)-N-PROC
9	2.48	1	2	0	0	806.46	806.40	N-(H)-N-PROC
10	2.57	1	1	2	0	895.5	895.44	S-(S-H)-N-ROC
11	2.61	1	2	1	0	952.51	952.46	N-(S-H)-N-ROC
12	2.64	1	3	0	0	1010.53	‎ 1009.48	H-N-(N)-N-ROC
13	2.82	1	2	1	0	952.51	952.46	N-(F)-H-N-ROC
14	2.87	2	2	0	0	969.5	968.46	-
15	2.98	1	1	0	1	894.48	894.42	S-(H)-N-PROC
16	3.05	1	3	1	0	1156.59	1155.54	S-H-N-(N)-N-PROC
17	3.28	2	2	0	0	968.52	968.46	H-(H-N)-N-PROC
18	3.41	1	2	2	0	1098.59	1098.52	N-(F)-H-(F)-N-PROC
19	3.69	3	1	0	0	927.1	927.43	-
20	3.73	2	2	1	0	1114.59	1114.51	H-N-(F-H)-N-PROC
21	3.78	2	2	1	0	1114.59	1114.51	H-N-(F-H)-N-PROC
22	3.85	2	2	0	1	1259.64	1259.55	S-H-N-H-N PROC
23	3.89	2	2	1	0	1114.59	1114.51	H-N-(F-H)-N-PROC
24	3.97	2	2	1	0	1114.58	1114.51	N-H-N-(F)-H-PROC
25	4.18	3	2	0	0	1130.58	1130.51	-
26	4.31	2	2	2	0	1260.65	1260.57	F-H-N-(F-H)-N-PROC
27	4.40	2	2	1	1	[703.00]^2+^	[703.31]^2+^	F-H-N-(S-H)-N-PROC

The O-glycans displayed a heterogenous mixture of neutral and acidic oligosaccharides and most of the structures were fucosylated. The MS/MS fragmentation data of procainamide labelled O-glycans contained diagnostic fragment ions characteristic of core type or blood group epitope consistent with previously reported data [29, 21, 48, 49]. The O-glycans carried the blood group H-epitopes Fucα1-2Galβ1 (diagnostic structure: Fuc-Hex) which is usually modified by a single monosaccharide residue (Galα1-3- or GalNAcα1-3-) and determines ABO blood groups [31, 50, 51]. The O-glycans found in this study were released from whole saliva from an individual of blood group A. The terminal structures of glycans 13 and 18 (Fig 5, Table 1) have a HexNAc attached to (Fuc)-Hex-HexNAc-Proc (blood group H-epitope), suggesting expression of blood group A antigen GalNAcα1-3(Fucα1–2)Galβ1- on saliva glycoproteins. The presence of ABO blood group specific epitopes matching the blood group status suggest that the saliva donor is secretor.

**Fig 6:**
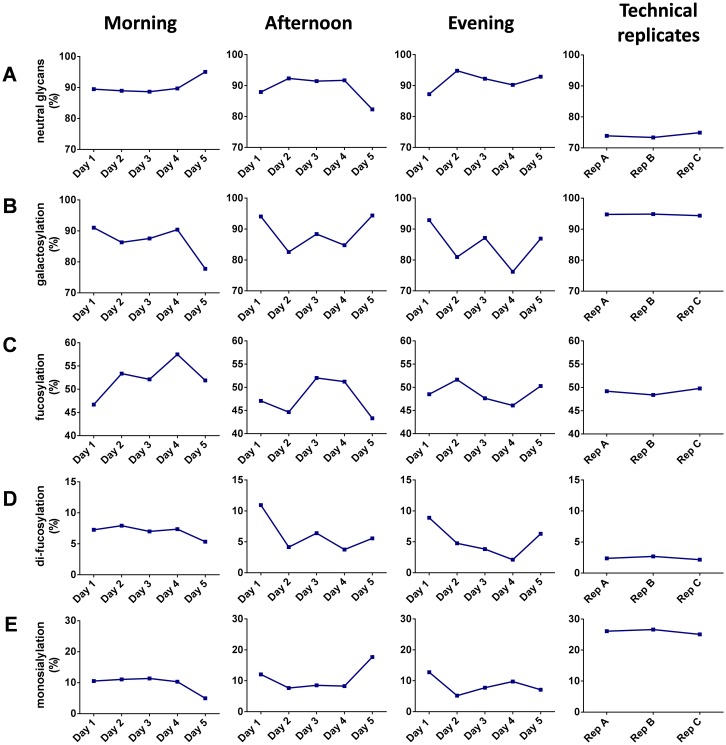
Saliva O-glycosylation variation during collection. Several differences in saliva O-glycosylation between different collection times and days can be observed with respect to (A) neutral glycans, (B) galactosylation, (C) fucosylation, (D) difucosylation, (E) monosialylation. Data for the technical replicates corresponds to the saliva reference sample collected from a single individual and analysed during evaluation of method repeatability and robustness.

Derived glycosylation features such as galactosylation and fucosylation were calculated for the selected O-glycan peaks to analyse the range of variability. Firstly, we compared levels of galactosylation (Fig 6B). A pronounced decrease in galactosylation (from 91% on the first day to 78% on the fifth day) was observed for the samples collected in the morning. For the samples collected in the afternoon or evening, variation was also high (between 83% and 94% for afternoon collection and 76% and 93% for evening collection).

Next we looked at changes in fucosylation for the samples collected three times a day during a 5 days period (Fig 6C). High biological variation was observed both intra-day and inter-day with fucosylation levels ranging from 43% to 57%.

We also looked at changes in sialylation. Significant variation (between 5% and 13%) between samples collected at different points of the day during the 5 days period was observed (Fig 6E).

The peak alignment across all HILIC chromatograms from two individuals shows that the individuals differ in relative peak abundance, while displaying similar variation within each peak (S3C Fig).

The main aim of this study was to analyse and compare entire O-glycan populations released form whole human saliva samples collected 3 times a day over a 5 days period. Our results highlighted that there is extensive natural variation in the salivary O-glycosylation. Both the intra-day and the inter-day variation appeared to be high.

We used whole human saliva because the wide spectrum of compounds (including glycoproteins) present in saliva may provide information for potentially important biomarkers and it has been reported that 30% of blood proteins are also present in saliva [52]. Also changes in O-glycans have been associated with different disease states [53, 54]. It has been previously published that the variation in salivary MUC5B glycosylation is high and dependent on blood group or secretor status [31]. Likewise, one may expect that salivary O-glycosylation, along with dynamic changes in composition of other salivary analytes may depend on diet, lifestyle and inflammatory responses in the mucus layer, although additional studies will be necessary to study this. One of the biggest advantages of saliva is easy collection, storage and transportation. Donation is non-invasive, stress-free and it does not require highly-trained personnel [55].

## Conclusions

Our study showed that the composition and relative abundance of the O-glycans in human saliva changes considerably during the day. This is an interesting observation and should be taken into account when evaluating salivary O-glycosylation *e*.*g*. for its diagnostic biomarker potential.

The changes in the specific O-glycan types within one day as well as between days may be caused by changes in the salivary concentrations of specific proteins, but may likewise be attributed to changes in protein-specific glycosylation profiles. It may, therefore, make sense to follow-up the current study and assess the glycosylation of one of the mucins abundantly present in saliva with respect to its longitudinal robustness and biomarker potential. Mucins found in saliva have high turnover rates and respond quickly to altered conditions within the body [32, 56]. Mucins and their O-glycans affect a range of aspects of oral health and their changes in the response to the infection and inflammation [32].

## Supporting Information

S1 Table Summary of O-glycan structures from fetuin with GU values, average relative abundance and mass values. (PDF)

S1 Fig MS/MS characterization of procainamide labelled fetuin O-glycan following online HILIC-UHPLC. (a) MS/MS characterization of the glycan eluting at retention time equivalent to that of GU 4.91. (b) Fragmentation scheme of the [M+2H]^2+^ ion at *m/z* 775.84. The nomenclature used for fragmentation is based on literature [58].
(PDF)

S2 Fig Fluorescence, MS and MS/MS traces of saliva O-glycan patterns. One sample, collected from individual 1 at day 1 in the morning, was used for in-depth analysis. (a) HILIC-FLR trace (FLR); (b) base peak chromatogram (BPC); (c) total ion chromatogram (TIC).
(PDF)

S3 Fig Comparison of HILIC-UHPLC O-glycan profiles of saliva samples collected from two individuals. The 7 peaks labelled with asterisks correspond to signals suitable for alignment and integration. (a) Saliva sample from individual 1 collected at day 1 in the morning; (b) Saliva sample from individual 2 collected at day 1 in the morning; (c) Comparison between individual 1 and 2 of the variation across time within 7 O-glycans measured by HILIC-UHPLC. After chromatogram alignment, the signals were integrated and the obtained areas normalized to the total area sum. The individuals show to differ in relative peak abundance, while displaying similar variation within each peak.
(PDF)
