# Supplementary material for: Variation of Human Salivary O-Glycome
Source: PLoS One. 2016 Sep 9;11(9):e0162824. doi: 10.1371/journal.pone.0162824 (PMC5017618; doi:10.1371/journal.pone.0162824)
Supplement: S1 Table — (PDF) [file pone.0162824.s001.pdf]

UHPLC Peak Id	Average		Structure	HILIC-UHPLC-ESI-MS and MS/MS							
	GU (PROC)	% Area		Composition			$[m/z]^{+}$ calculated	$[m/z]^{2+}$ calculated	$[m/z]^{+}$ registered	$[m/z]^{2+}$ registered	[m/z] characteristic B and Y fragment ions (composition)
				Hex (H)	HexNAc (N)	Neu5Ac (S)					
**1**	**1.93**	**6.80**	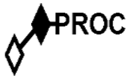	1	1	0	603.32	302.17	603.35	nd	441.30 (N1-PROC)
**2**	**2.18**	**16.15**	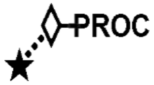	1	0	1	691.34	346.17	691.36	nd	292.12 (S); 400.27 (H1-PROC)
**3**	**2.50**	**62.82**	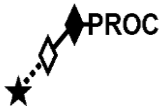	1	1	1	894.42	447.71	894.41	nd	441.31 (N1-PROC); 603.34 (H1N1-PROC)
**4**	**3.13**	**1.59**	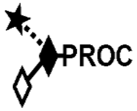	1	1	1	894.42	447.71	894.41	nd	441.32 (N1-PROC); 603.37 (H1N1-PROC)
**5**	**3.90**	**10.42**	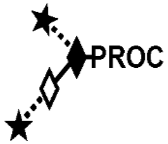	1	1	2	1185.51	593.26	1185.54	593.30	292.13 (S); 441.26 (N1-PROC); 454.16 (HS); 603.33 (H1N1-PROC); 732.36 (N1S1-PROC); 894.38 (H1N1S1-PROC)
**6**	**4.91**	**2.22**	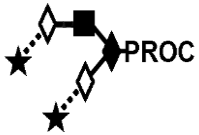	2	2	2	1550.65	775.83	nd	775.84	292.12 (S); 366.18 (H1N1); 441.28 (N1-PROC); 454.16 (H1S1); 603.36 (H1N1-PROC); 644.34 (N2-PROC); 657.24 (H1N1S1); 806.40 (H1N2-PROC); 894.39 (H1N1S1-PROC); 968.38 (H2N2-PROC); 1097.46 (H1N2S1-PROC); 1259.51 (H2N2S1-PROC)
