# Supplementary figures and images for: Variation of Human Salivary O-Glycome
Source: PLoS One. 2016 Sep 9;11(9):e0162824. doi: 10.1371/journal.pone.0162824 (PMC5017618; doi:10.1371/journal.pone.0162824)

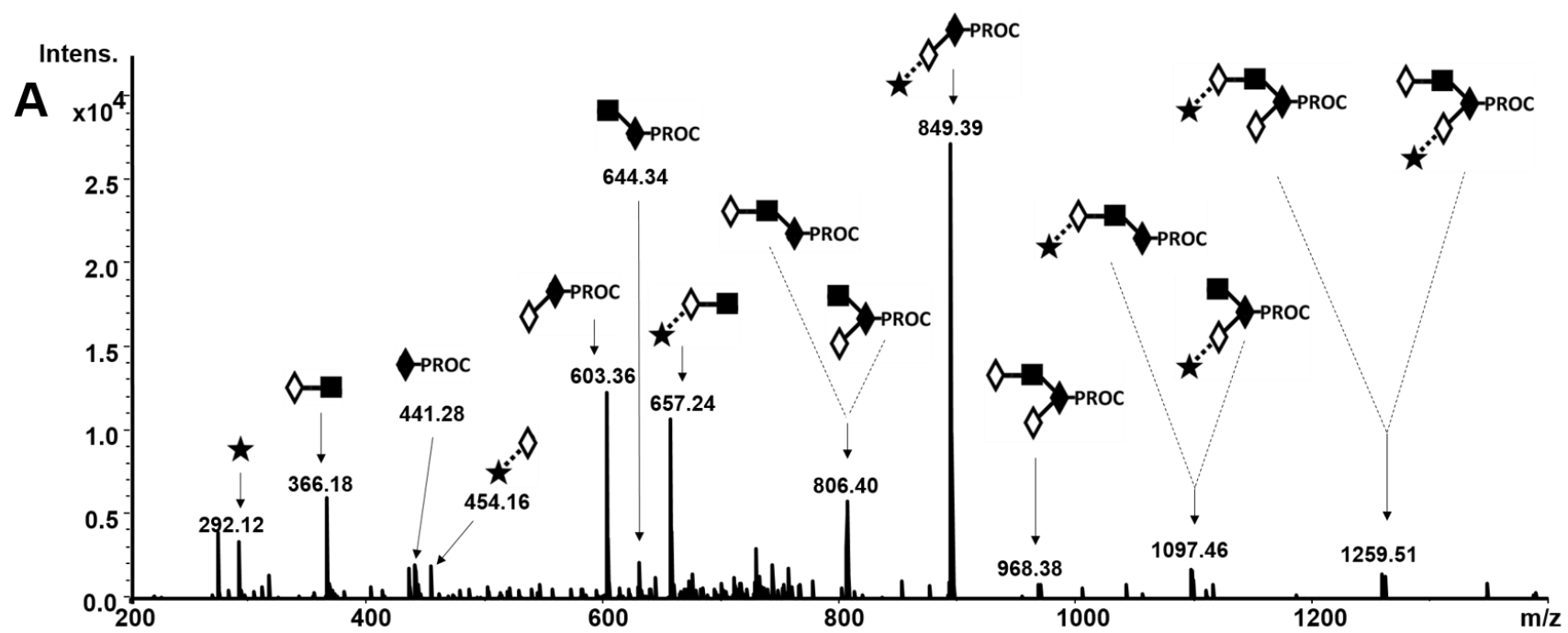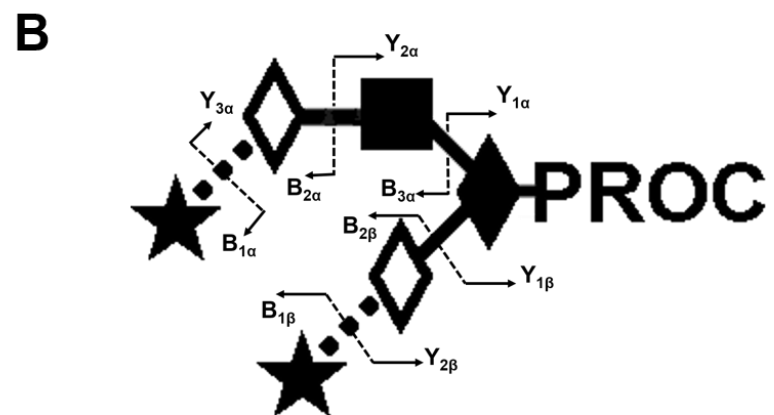

Supplement: S1 Fig — (a) MS/MS characterization of the glycan eluting at retention time equivalent to that of GU 4.91. (b) Fragmentation scheme of the [M+2H]2+ ion at m/z 775.84. The nomenclature used for fragmentation is based on literature [58]. (PDF) [file pone.0162824.s002.pdf]

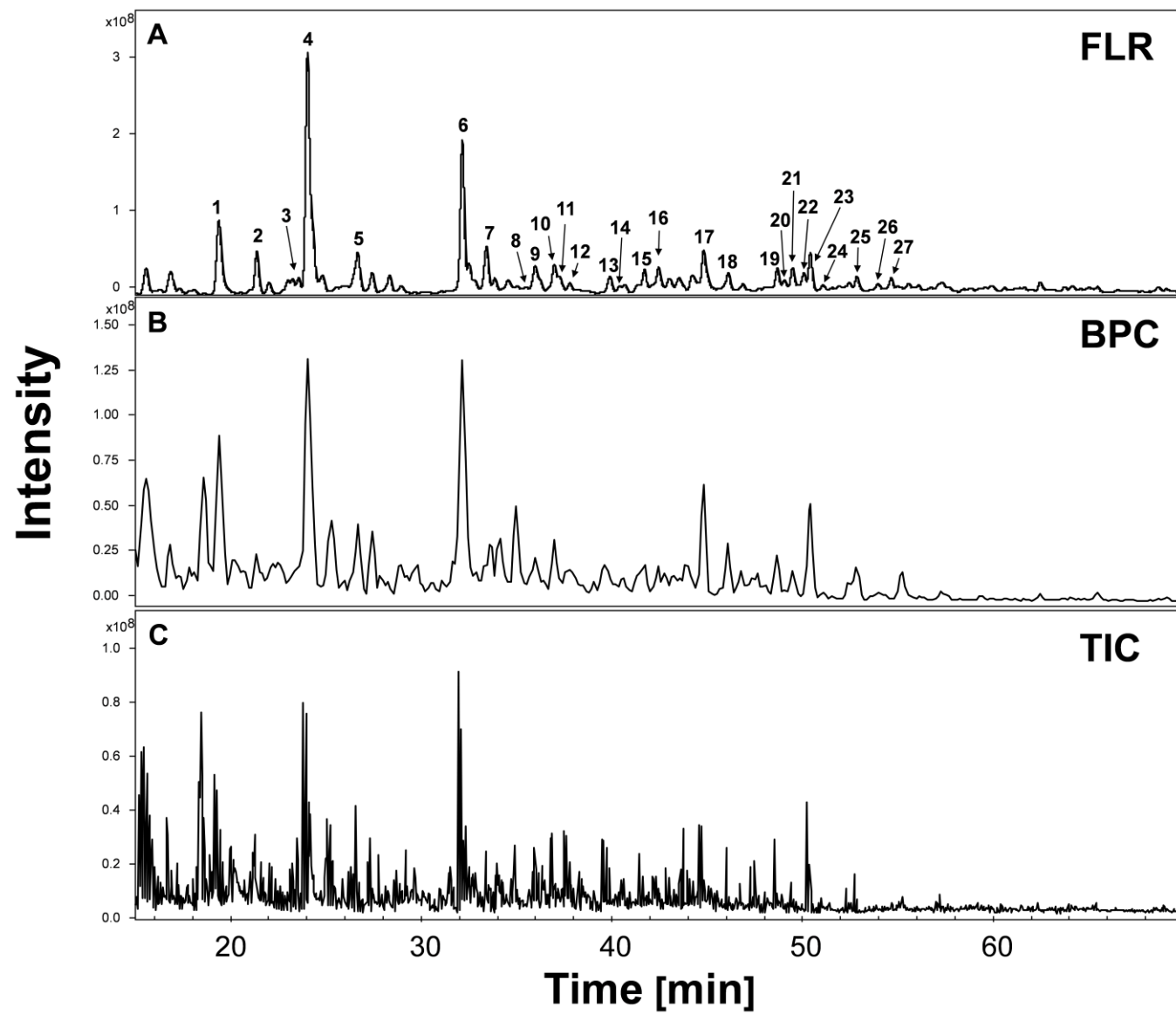

Supplement: S2 Fig — One sample, collected from individual 1 at day 1 in the morning, was used for in-depth analysis. (a) HILIC-FLR trace (FLR); (b) base peak chromatogram (BPC); (c) total ion chromatogram (TIC). (PDF) [file pone.0162824.s003.pdf]
