# Supplementary material for: Variation of Human Salivary O-Glycome
Source: PLoS One. 2016 Sep 9;11(9):e0162824. doi: 10.1371/journal.pone.0162824 (PMC5017618; doi:10.1371/journal.pone.0162824)
Supplement: S3 Fig — The 7 peaks labelled with asterisks correspond to signals suitable for alignment and integration. (a) Saliva sample from individual 1 collected at day 1 in the morning; (b) Saliva sample from individual 2 collected at day 1 in the morning; (c) Comparison between individual 1 and 2 of the variation across time within 7 O-glycans measured by HILIC-UHPLC. After chromatogram alignment, the signals were integrated and the obtained areas normalized to the total area sum. The individuals show to differ in relative peak abundance, while displaying similar variation within each peak. (PDF) [file pone.0162824.s004.pdf]

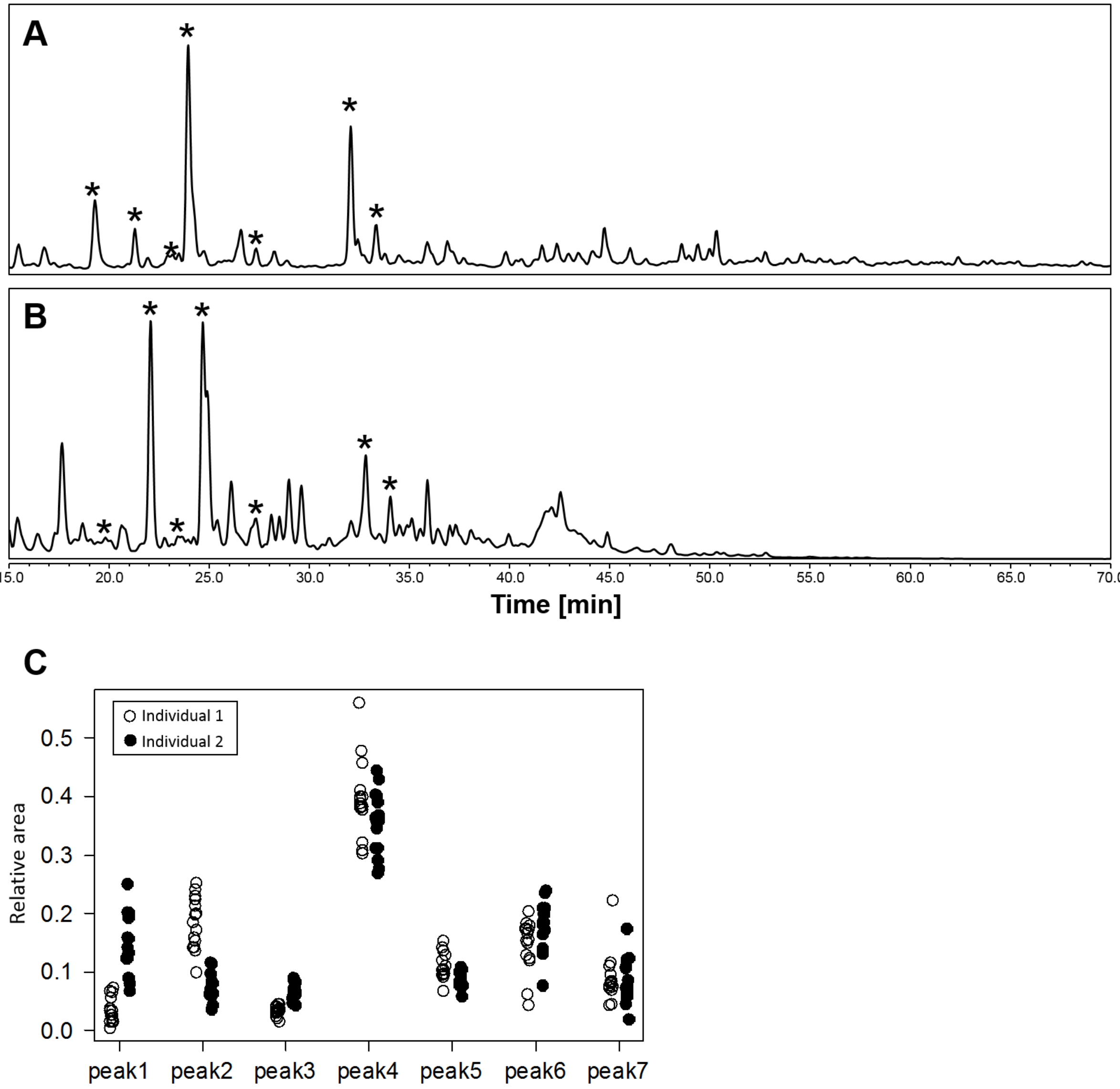

A
B
15.0
20.0
25.0
30.0
35.0
40.0
45.0
50.0
55.0
60.0
65.0
70.0
Time [min]
C
Individual 1
Individual 2
Relative area
0.0
0.1
0.2
0.3
0.4
0.5
peak1
peak2
peak3
peak4
peak5
peak6
peak7
